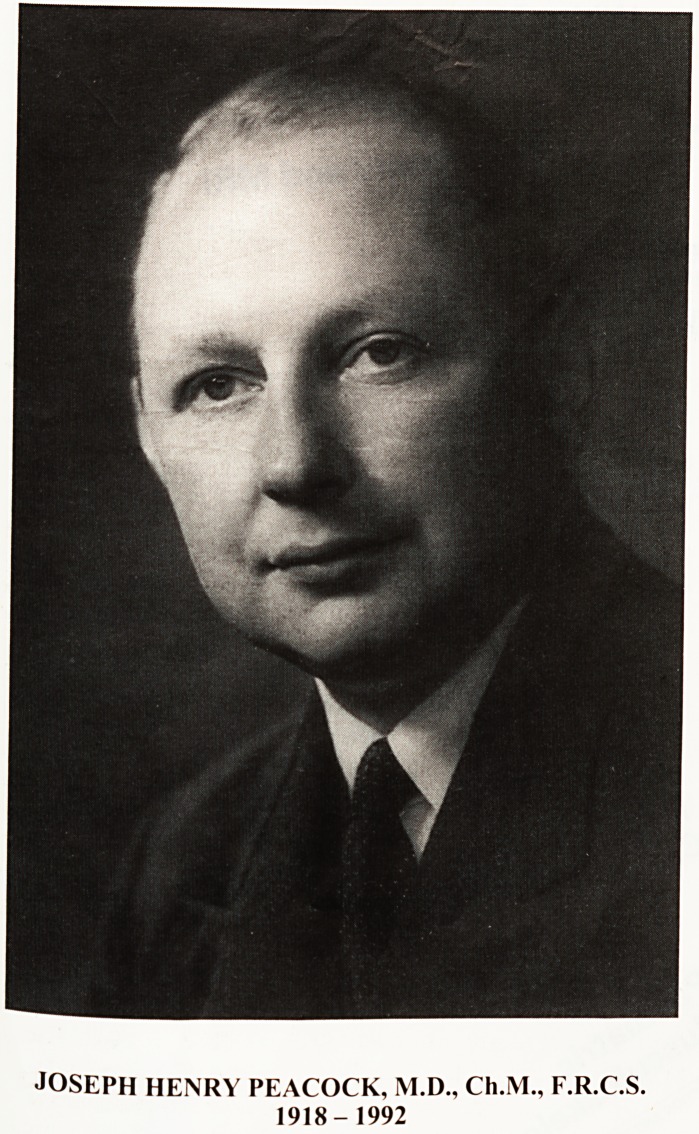# Professor J H Peacock

**Published:** 1992-12

**Authors:** 


### Obituary

**JOSEPH HENRY PEACOCK, M.D., Ch.M., F.R.C.S.**
**1918 – 1992**

Professor Joseph Peacock died suddenly at his home on the 6th of May. His father was General Manager of the Great Western Railway and received the OBE. He was educated at Bristol Grammar School, Reading School and Birmingham University. At Birmingham University he began initially to read Dentistry but after his first year changed over to Medicine. He graduated MB ChB in 1941 and also obtained the MRCS, LRCP. Initial house appointments were HS to Prof. Andrade at Birmingham and Orthopaedic HS at Stanmore Orthopaedic Hospital. From 1942 - 1947 he served in the RAMC beginning with a period as RMO to the 19th Royal Fusiliers. Later he served in Military Hospitals in India and Malaya and ended the war as an orthopaedic specialist. On demobilisation he became a Demonstrator in Anatomy at the University of Birmingham and in 1949 obtained the FRCS and was appointed Surgical Registrar at the Bristol Royal Infirmary to Professor Milnes Walker. In 1950 he was awarded a Rockefeller travelling Fellowship at Ann Arbor, Michigan, investigating the role of nordrenaline in Raynaud's Disease. In 1953 he was appointed Lecturer in Surgery at Bristol University and Consulting Surgeon at Bristol Royal Infirmary. In 1954 he was awarded the Jacksonian Prize for his research into Raynaud's Disease and in 1956 a Hunterian Professorship at the RCS. In 1960, he became a member of the SW Regional Hospital Board and was Chairman of their Research Committee. In that year he was also an Arris and Gale Lecturer at the RCS. In 1965 he was appointed a Reader in Surgery and in 1967 was awarded a Jacksonian Prize for the second time, an unusual achievement.

In addition to Raynaud's Disease his early research work also included the preservation of arterial grafts by freeze drying. In the Department of Surgery Milnes Walker's special interest was in portal hypertension and liver surgery and this was continued by his successor Atholl Riddell. It was a natural development that liver transplantation should become the focus of attention and Joe Peacock took up this challenge. In the late 1960s animal work began in association with the University department of Veterinary Surgery at Langford using the pig as the experimental animal. At this time success in the human had not been achieved. The pig proved to be a satisfactory animal model, much useful work was done developing the basic techniques and a large number of original papers emerged from the department. This pioneer work attracted a succession of young surgeons from South Africa, John Terblanche, Ed Immelman and David Dent. Success with the human was not achieved however, largely due to the difficulty in transporting donor livers in the short space of time required. Their work was overtaken in this country by Roy Calne at Cambridge who performed the first successful human liver transplantation in Britain in 1968 and who set up transport by helicopter and police motor cycle teams. In 1972 his devotion to research was recognised by the creation of a personal chair of Surgical Science. In 1975 he represented the University on the General Medical Council for over ten years and was chairman of the Overseas Subcommittee. He was a founder member of the Surgical Research Society and was also a member of the Vascular Surgical Society and the European Society of Surgical Research. He was a member of the Court of Examiners of the Royal College of Surgeons both for the Primary and for the Final FRCS. He was also sometime external examiner in Surgery for the Universities of Birmingham, London, Wales, Liverpool, Ghana and Sudan. In spite of all these activities he continued to exercise an overall supervision of the research programme of the Department of Surgery and to give advice and help to those embarking on new research. He was a warm and kindly man with a quiet sense of humour, preserving an old world courtesy, deliberate and calm in the operating theatre. Though specifically devoted to research his interest in clinical surgery remained firm and he continued to act as a full time member of the consultant staff. His grasp of surgery in all its aspects and his wide knowledge was most noticeable through his contributions at meetings, especially the weekly professorial rounds of the department of surgery rotating through the Bristol Hospitals.

In his youth he played squash for his county and hockey for Birmingham University. He always had an interest in the technical aspects of radio and developed this in retirement. He took a course and diploma in Electronics at Bristol Polytechnic lasting a year. He later acquired a license as a Radio Operator and became a 'Radio Ham'. with his own call sign. He was a member of an English Net, regularly spoke to people all over the world and at times corresponded with a net of New York doctors.

In 1950 he married Gillian Pinckney, at the time a medical student at Bristol University, a keen horsewoman and now prominent in the movement for Riding for the Disabled. Their beautiful home in the Old Manor at Ubley was the centre of their happy married life and the garden a hobby for both of them. He is survived by a son and a daughter.

M.G.W., J.P.M.

## Figures and Tables

**Figure f1:**